# 

**Published:** 1889-06

**Authors:** 


### Visit of the British Medical Association to Bristol.

---

In view of the probable visit of the British Medical Association to Bristol in 1891, the following have been appointed as a Committee to endeavour to render the Museum as perfect as possible:—

Dr. SHINGLETON SMITH, *Chairman.*

Dr. BARCLAY BARON.
Mr. DOBSON.
Dr. E. LONG FOX.
Dr. HARRISON.
Mr. HARSANT.
Dr. AUST LAWRENCE.
Dr. BARRETT ROUÉ.
Dr. SHAW.
Dr. MARKHAM SKERRITT.
Mr. GREIG SMITH.

Dr. WALDO.

Dr. J. MICHELL CLARKE,
Mr. DACRE,
Dr. PARKER,
Mr. G. MUNRO SMITH,
*Honorary Secretaries.*

This Committee asks for help from all readers of *The Bristol Medico-Chirurgical Journal* and others; and will be glad to receive Specimens, Casts, Plates, Photographs, etc., for the Annual Museum. All contributions of this kind will be taken care of and returned at the wish of the sender. They should be addressed to Mr. G. MUNRO SMITH, Medical School, Bristol.

### PUBLICATIONS RECEIVED.

From G. Leuzinger & Filhos, Rio de Janeiro:
*De la Lobéline dans la thérapeutique de l'Asthme.* Par le Dr. Silva Nimes. 1889.
From John Carnrick, New York:
*The Dietetic Gazette.* March, 1889.
From 95 William Street, New York:
*The International Journal of Surgery.* March, April, 1889.
From P. Blakiston, Son & Co., Philadelphia:
*The Polyclinic.* March—April, 1889.
From F. A. Davis, Philadelphia:
*Lectures on Nervous Diseases.* By Ambrose L. Ranney, A.M., M.D. 1888.
*The Physiology of the Domestic Animals.* By Robert Meade Smith, A.M., M.D. 1889.
From 3860 Pine Street, St. Louis:
*The Alienist and Neurologist.* April, 1889.
From Feret, Bordeaux:
*Annales de la Polyclinique de Bordeaux.* No. 1. Janvier, 1889.
From 5 Avenue de l'Opéra, Paris:
*Annales d'Orthopédie et de Chirurgie practiques.* Nos. 6—11. 1889.
From 26 Rue Houdan, Sceaux:
*La Médecine hypodermique.* No. 4. 1889.
From Eugen Grosser, Berlin:
*Therapeutische Notizen der Deutschen Medizinal-Zeitung.* Herausgegeber Dr. Julius Grosser. 1880—1889. Heft. I., II., 1889.
From John Wright & Co., Bristol:
*Elementary Bandaging and Surgical Dressing.* By Walter Pye. Third Edition. 1889
*Massage and Electricity.* By Thomas Stretch Dowse, M.D. n.d.
From Young J. Pentland, Edinburgh:
*The Treatment of Epilepsy.* By William Alexander, M.D. 1889.
*The Causes and Treatment of Abortion.* By Robert Reid Rentoul, M.D. 1889.
*Studies in Clinical Medicine.* By Byrom Bramwell, M.D. Vol. I., Nos. 1—3. 1889.
*Diseases of the Eye.* By George A. Berry, M.B. 1889.
From George Bell & Sons, London:
*Therapeutics Founded on Antipraxy.* By William Sharp, M.D., F.R.S. 1886.
From Cassell & Company, Limited, London.
*Elements of Histology.* By E. Klein, M.D., F.R.S. 1889.
From J. & A. Churchill, London:
*Instruments and Appliances required in Operations.* By A. W. Mayo Robson. 1889.
*The Germ-Theory of Disease.* By Francis W. Clark. 1888.
*La Méthode antiseptique chez les Anciens.* Par A. Anagnostakis. 1889. (Karl Wilberg, Athens).
*What will Trained Nurses gain by joining the British Nurses' Association?* [By Eva C. E. Lückes.] 1889.
*An Essay on Asphyxia.* By George Johnson, M.D., F.R.S. 1889.
*On Rhinophyma.* By Balmanno Squire, M.B. 1889.
From Charles Griffin & Company, London:
*A Surgical Handbook.* By Francis M. Caird, M.B., and Charles W. Cathcart, M.B. 1889.
From H. K. Lewis, London:
*Practice of Medicine.* By Angel Money, M.D. 1889.
*Osteo-Arthritis.* By John Kent Spender, M.D. 1889.
*The British Journal of Dermatology.* April—June, 1889.
*Lateral Curvature of the Spine.* By Bernard Roth. 1889.
*Strathpeffer Spa.* By Fortescue Fox, M.D. 1889.
*Tooth Extraction.* By John Gorham. Third Edition. 1889.
*The Circulation through Diseased Hearts.* By Herbert Davies, M.D. Edited by Arthur Templer Davies, B.A., M.B. 1889.
From E. Truelove, London:
*Home Rule and Federation.* By A Doctor of Medicine. 1889.
From H. Vickers, London:
*The Tocsin.* No. 4. April, 1889.
From John Heywood, Manchester:
*Defects in Plumbing and Drainage Work.* Described by Francis Vacher. 1889.

*Cases for binding, price 7d. each, may be had of the Publisher,*
J. W. ARROWSMITH, *Quay Street, Bristol.*

Royal Arsenal,

Woolwich,

*June 10th*, 1888.

I am desirous of bringing to the notice of Civil and Military Surgeons a first dressing for wounds, which I have designed under the name of a "First Field Dressing." As is well known, the subsequent course of a wound depends so much on the treatment first applied to it, that it has been well said that the fate of the wounded man lies in the hands of the surgeon who first attends him. What should be the most suitable first dressing for a wound, whether received in action or as the result of an accident in civil life, has been the subject of much discussion among surgeons, military and civil, and though all are agreed as to the enormous advantages of antiseptic first dressings to wounds, yet there exists a considerable divergence of opinion as to what form these should take; all, however, holding that a first dressing should be simple, light, complete in its requirements, and equally useful in the hands of non-professional as in those of a professional attendant. This dressing seems to meet this want. It consists of layers of Alembroth Wool, enclosed in Alembroth gauze, forming a pad 5 in. long and 3½ in. wide. To this an Alembroth bandage, about 1½ yds. long, is attached about its centre, and forms, with the gauzed wool, a pocket in which is placed a small gauze bag of Iodoform. A safety pin completes the dressing. The whole is enclosed in air-proof parchment with directions as to use, viz., to dust out the Iodoform from the bag on to the wound, and apply the pad with attached bandage and pin. Complete it weighs an ounce. For field purposes, a triangular surgical scarf is found useful, therefore the maker is instructed to send out one with every dozen packets. It is prepared of the same special material as the bandage, and measures extreme length 46 in., width 23 in. It will be observed that all these materials possess the most powerful, and, I may add, the most approved, antiseptic properties. I would, therefore, submit this as a First Dressing to wounds immediately on their occurrence, and, though primarily intended for wounds received on the battle field, it will be found to be a most suitable first dressing for the various wounds met with in civil life. It is simple in design, clean to handle, and can be applied by anyone, whether conversant with antiseptic surgical dressings or otherwise. Possessing no volatile principles the dressing will always retain its antiseptic properties, it will not be affected by climate, and is preserved from damp, etc., by the special parchment cover. I submit it as the most complete antiseptic occlusive First Dressing for a wound. I would further point out that should a wound require an immediate washing, an approximate corrosive sublimate solution can be made by simply taking out some of the Alembroth wool and placing it in water. To ensure economy in price and correct quality of the materials used in these dressings, I have instructed Mr. John Milne, of Ladywell (who prepares Professor Lister's Antiseptic materials). to manufacture these dressings.

ULICK J. BOURKE,
Surgeon-Major,
Army Medical Staff.

